# Prognostic and predictive factors associated with ipilimumab-related adverse events: a retrospective analysis of 11 NCI-sponsored phase I clinical trials

**DOI:** 10.18632/oncotarget.27537

**Published:** 2020-04-21

**Authors:** Aman Chauhan, Tanvir Kabir, Jianrong Wu, Jing Wei, Mary Cook, Charles A. Kunos

**Affiliations:** ^1^Division of Medical Oncology, University of Kentucky, Lexington, KY, USA; ^2^College of Medicine, University of Kentucky, Lexington, KY, USA; ^3^Markey Cancer Center, Biostatistics and Bioinformatics Shared Resource Facility, University of Kentucky, Lexington, KY, USA; ^4^Department of Statistics, University of Kentucky, Lexington, KY, USA; ^5^Cancer Therapy Evaluation Program, National Cancer Institute, Bethesda, MD, USA

**Keywords:** immunotherapy, toxicity, co-administered agents, metastatic melanoma, immune checkpoint inhibitors

## Abstract

Background: We review factors impacting ipilimumab-associated adverse events through the experience from National Cancer Institute (NCI)-sponsored phase I immunotherapy clinical trials.

Materials and Methods: Attributable ipilimumab-related adverse events from NCI-sponsored phase I immunotherapy clinical trials were queried retrospectively by anonymized patient experience reports for observed adverse events like decreased hematological cell counts, blood electrolytes or proteins, or reduced patient performance status. The prevalence of ipilimumab-related toxicity was associated by patient to the duration of ipilimumab exposure, radiographic responses, progression-free survival, and overall survival.

Results: 373 patients from 11 phase 1 ipilimumab clinical trials were analyzed. Patients experiencing at least one grade 3 or 4 adverse event associated with observed radiographic response were included. The average number of grade 3/4 adverse events in responders was 1.167 versus 0.645 in non-responders (*p* = 0.001). Patient performance status did not significantly impact observed toxicity grade. Pretherapy lymphocyte count or chemistries were not associated with ipilimumab-associated toxicity. The number of agents combined with ipilimumab on trial was associated with average number of grade 3/4 toxicities–ipilimumab monotherapy (0.631) versus ipilimumab + 1 agent (0.877) versus ipilimumab + 2 agents (1.408) (*p* = 0.014). Number of low grade (grade 1/2) toxicities was associated with duration of treatment, Pearson correlation coefficient *r* = 0.456 (*p* < 0.0001); whereas the number of high grade (grade 3/4) toxicities was not, *r* = 0.032 (*p* = 0.546).

Conclusions: Ipilimumab-attributed grade 3/4 toxicity was associated with therapeutic response. The number of co-administered agents added to ipilimumab significantly raised the likelihood of toxicity. Extended duration of treatment increased the incidence of low but not high-grade toxicity.

## INTRODUCTION

Cancer clinical trials offer the best data to study, treat, and cure cancer. Over 1,400 clinical trials are actively recruiting cancer patients to treatments that involve immunotherapy with at least one anticancer agent, with ipilimumab studied in about 500 of those trials [1]. The immune checkpoint inhibitor ipilimumab inhibits cytotoxic T-lymphocyte associated protein 4 (CTLA4 or CD152) and renders exposed cancer cells sensitive to T-cell mediated destruction [2]. The number of ipilimumab users in cancer clinics has expanded substantially and ipilimumab currently serves as one of the most often combined cancer immunotherapy agents against cancer. The best known ipilimumab-associated adverse event or toxicity experience arises from human clinical trials evaluating it against skin cancer melanoma [3]. Limited data are available for biological or clinical markers that might predict ipilimumab-attributed toxicity.

In this retrospective article, we collect and analyze ipilimumab-attributed toxicity from 11 National Cancer Institute (NCI)-sponsored phase 1 trials evaluating the agent in a variety of adult solid and hematological cancers with the aims of associating patient and clinical factors with engendered ipilimumab-attributed toxicity and treatment outcomes.

## RESULTS

Data from 373 patients from 11 phase I ipilimumab clinical trials were available for the analysis. Median age of the study cohort was 55 years and 68% (*n* = 253) were males. ECOG performance status was available in all but nine patients. 222 patients (61%) had ECOG performance status of 0, and 142 (38%) had ECOG performance status of 1 or 2 at the time of enrollment. No significant differences in grade 1 or 2 (here deemed low grade), or, grade 3 or 4 (high grade) adverse events were identified in the study cohorts based on ECOG performance status (0 versus 1 or 2). Patients with ECOG performance status 3 or 4 were not eligible for these clinical trials.

We also looked into association between toxicity and pretreatment albumin (a surrogate for baseline nutritional status), lactate dehydrogenase (LDH; a surrogate marker for tumor bulk) and lymphocyte count (hypothesis being higher baseline lymphocyte count might result in higher autoimmune toxicities). Pretreatment albumin was available for analysis in 92 patients, pretreatment LDH was available in 90 patients and pretreatment lymphocyte count was available in 88 patients. Pretreatment lymphocyte count, LDH or albumin was not predictive of increased ipilimumab associated toxicity (Table 1).

**Table 1:** Patient demographic and prognostic variables, toxicity and outcome results

Variable	*N* (%)	All grade Mean (STD)	Grade 3 + Grade 4 Mean (STD)
Age (continuous)	*N* = 373	*r* = 0 .089 (*p* = 0.084)	*r* = 0.062 (*p* = 0.232)
Mean	52		
Median	55		
Age (categorical)			
≤ 55	187	6.02 (6.15)	0.717 (1.25)
> 55	186	6.69 (5.15) (*p* = 0.029)	0.833 (1.38) (*p* = 0.398)
Sex			
Female	120 (32%)	6.01 (6.83)	0.625 (1.19)
Male	253 (68%)	6.52 (5.31) (*p* = 0.103)	0.846 (1.37) (*p* = 0.111)
ECOG PS			
0	222 (61%)	6.76 (5.72)	0.748 (1.31)
1 and 2	142 (38%)	5.78 (5.67) (*p* = 0.048)	0.803 (1.370) (*p* = 0.590)
Missing (excluded)	9 (2%)		
Number of agents			
1	243 (65%)	5.61 (5.57)	0.613 (1.05)
2	81 (22%)	6.62 (5.86)	0.877 (1.54)
3	49 (13%)	9.59 (4.73)	1.408 (1.84)
Overall chi-square test		*p* < 0.0001	*p* = 0.0136
Pairwise test (1 vs 2)		*p* = 0.146	*p* = 0.3174
Pairwise test (1 vs 3)		*p* < 0.0001	*p* = 0.0035
Pairwise test (2 vs 3)		*p* = 0.0002	*p* = 0.085
Pre-treatment albumin	92	*r* = 0.157 (*p* = 0.136)	*r* = 0.142 (*p* = 0.178)
Pre-treatment LDH	90	*r* = –0.289 (*p* = 0.006)	*r* = –0.132 (*p* = 0.213)
Pre-treatment lymphocyte count	88	*r* = –0.077 (*p* = 0.475)	*r* = –0.028 (*p* = 0.797)
Best response			
No response	273	6.01	0.645
CR+PR	54	9.27 (*p* < 0.0001)	1.167 (*p* = 0.001)
Disease progression			
Yes	192	5.01	0.578
No	135	8.74 (*p* < 0.0001)	0.948 (*p* = 0.039)
Duration of therapy	366	*r* = 0.456 (*p* < 0.0001)	*r* = –0.032 (*p* = 0.546)

Radiological responses were correlated with the grade of observed adverse events. Best radiological responses were analyzed and divided between two cohorts—stable disease (*n* = 273) versus complete or partial response (CR/PR) (*n* = 54). Grade 3 and 4 adverse events were associated with better radiological response (*p* = 0.001). The average number of grade 3 or 4 adverse events in the CR/PR cohort was 1.167, versus 0.645 in the non-responder cohort.

Of all patients, 243 (65%) were treated with only ipilimumab, 81 patients (22%) were treated with two agents including ipilimumab and 49 patients (13%) were treated with three agents including ipilimumab. Ipilimumab-associated grade 3 and 4 toxicity was directly associated with the co-administered number of study agents. The average number of grade 3 and 4 adverse events was 0.631 after ipilimumab alone, 0.877 after ipilimumab plus one other agent and 1.408 after ipilimumab plus two agents (*p* = 0.014).

Lastly, we looked into the association of low- and high-grade toxicities with regard to duration of ipilimumab treatment. Data on duration of therapy were available on 366 patients. Low grade (grade 1 or 2) toxicity was associated with the duration of treatment. The longer patients stayed on treatment, the higher the incidence of grade 1 or 2 adverse events as suggested by Pearson correlation coefficient (*r* = 0.456; *p* < 0.0001). The number of high-grade (grade 3 or 4) toxicity was not associated with the duration of treatment with ipilimumab (*r* = 0.032; *p* = 0.546). Grade 3 or 4 adverse events were noted in patients with both minimal exposure to drug as well as those who were on ipilimumab for a long duration (See Table 1).

## DISCUSSION

Although numerous studies evaluate the efficacy and toxicity of ipilimumab in melanoma, less data are available on ipilimumab-associated adverse events in other types of solid and hematological tumors. Furthermore, data regarding clinical and serum markers that may predict such adverse events has been limited. From among 373 patients participating in 11 ipilimumab immunotherapy trials sponsored by the NCI to treat various cancers, our study found most importantly and not unexpectedly that ipilimumab-attributed toxicity associated with the number of co-administered agents, and the prevalence of toxicity increased according to the number of agents in the ipilimumab combination (See Table 2). This finding can be put into context with a randomized double-blind phase III trial treating 945 metastatic melanoma patients taking/using/administering either ipilimumab and nivolumab together, or either ipilimumab or nivolumab individually/alone.

**Table 2:** Tumor types and study agents across 11 phase 1 clinical trials

Disease Type	Study Agents
Lung Adenocarcinoma	Ipilimumab
Ovarian Neoplasms	Gp-100 (Anti-Tumor Vaccine)
Chronic Lymphocytic Leukemia	GM-CSF
Mantle Cell Lymphoma	Dasatanib
Hodgkin’s Lymphoma	Cetuximab
Myeloma	Rituximab
Follicular Lymphoma	Prostvac (Oncolytic Virus)
Adenocarcinoma NOS	External Beam Radiation
Prostate Cancer	
Sarcoma	
Renal Cell Carcinoma	
Malignant Melanoma	
Neuroblastoma	
Osteosarcoma	
Soft Tissue Neoplasm	
Gastrointestinal Stromal Tumor	
Head and Neck Carcinoma	
Non-Hodgkin’s Lymphoma	
Myelodysplastic Syndrome	
Acute Myeloid Leukemia	

The prevalence of grade 3 or 4 adverse events was higher for the combination as compared to either agent alone. In this trial, 55% of patients treated with both ipilimumab and nivolumab experienced grade 3 or 4 adverse events, whereas 27% of patients after ipilimumab alone and 16% of patients after nivolumab had grade 3 or 4 adverse events. Despite an increase in overall survival after ipilimumab-nivolumab treatment, the relatively high prevalence of severe toxicities after combination treatment became a barrier to treatment. It is noteworthy that the resolution of severe immunotherapy-related adverse events was similar in all three patient treatment groups.

Various studies involving checkpoint inhibitor combination therapy are consistent with these findings. A retrospective analysis of approximately 7700 metastatic melanoma patients treated with ipilimumab and/or nivolumab showed that when used concurrently, their toxicity profile combines adverse events from each individual drug [4]. Another retrospective analysis of a phase I, II and III trial consisting of 448 patients with advanced melanoma treated with ipilimumab and nivolumab demonstrated higher levels of grade 3 and 4 adverse events (55%) compared to either agent alone [5]. Greater rates of toxicity have been reported even when immune checkpoint inhibitors have been used sequentially. For example, in a retrospective analysis of phase III studies, 40 patients with metastatic melanoma received nivolumab or pembrolizumab and then upon progression received ipilimumab [6]. 35% of patients from this phase III study developed grade 3–5 immune related adverse events. Three patients (7%) developed grade 3–5 pneumonitis leading to the death of one patient. A randomized phase II study consisting of 140 patients found clinical benefit when nivolumab was used sequentially with ipilimumab; however, it was also noted that the sequential combination was associated with a greater frequency of adverse events [7].

When testing ipilimumab concurrently with cytotoxic chemotherapy, the results were similar. In a phase III trial evaluating ipilimumab with or without dacarbazine in 502 patients with untreated metastatic melanoma, grade 3 and 4 adverse events were more frequent in ipilimumab plus dacarbazine (56%) compared to dacarbazine plus placebo (28%) [8]. The pattern of immune-related adverse events differed, with approximately 20% more hepatotoxicity and 2% less colitis in the ipilimumab plus dacarbazine group. In a phase III study treating 954 small cell lung cancer patients with either ipilimumab with etoposide and platinum or with chemotherapy alone, rates and severity of treatment-related adverse effects were similar between groups except more frequent diarrhea, rash and colitis occurred in the group treated with ipilimumab plus chemotherapy [9]. There was significantly higher treatment related discontinuation in the group with ipilimumab (18%) compared to the control group (2%). Even when evaluating ipilimumab in addition to radiotherapy, grade 3 and 4 adverse events may be significantly higher. In a phase III trial treating 799 patients with metastatic prostate cancer that had progressed after docetaxel treatment, patients were treated via radiotherapy with or without ipilimumab [10]. Three percent of patients treated with radiation therapy alone experienced grade 3 or 4 adverse events, while 26% treated with radiation plus ipilimumab experienced grade 3 or 4 adverse events. Only 4 deaths occurred (1%), all in the radiation plus ipilimumab group. Our results are consistent with current data. Grade 3 and 4 adverse events escalate rather rapidly when ipilimumab is combined with additional treatment. This escalation is more pronounced with three or more combination agents.

Our data also showed that grade 3 and 4 toxicity predicted response to treatment and was associated with higher complete and partial responses. This would be theoretically expected, as increased immune-related adverse effects should be elevated when the immune system is stimulated. However, conflicting evidence in past studies both support and refute this finding. In a retrospective analysis of 86 patients treated with ipilimumab, 42% of patients suffered from autoimmune toxicity and occurrence of toxicity correlated with clinical benefit [11]. Likewise, in a retrospective study consisting of 129 metastatic melanoma patients treated with ipilimumab, the development of any toxicity during treatment was associated with higher progression-free survival [12]. Multivariate analysis showed that absence of toxicity was a prognostic factor for poor overall survival (OS) and progression-free survival. In an analysis of 198 patients in Germany treated with ipilimumab, the occurrence of immune-related adverse events correlated significantly with response to treatment and prolonged OS [13].

On the other hand, a retrospective analysis of 298 patients with melanoma treated with ipilimumab at Memorial Sloan Kettering Cancer Center between 2011 and 2013 demonstrated that OS and time to treatment failure were not affected by the occurrence of immune-related adverse events or treatment with corticosteroids [14]. No differences in OS or time to treatment failure were found when patients were stratified by immune related adverse events of any grade. These findings were consistent with another retrospective analysis of 855 patients in Italy treated with ipilimumab, in which the investigators concluded that the frequency of immune related adverse events was not associated with response to ipilimumab [15]. Similarly, in an analysis of 209 patients with advanced melanoma treated with ipilimumab, the occurrence of adverse events did not correlate with clinical benefit to ipilimumab [16]. Thus, there is conflicting evidence in the literature regarding a correlation between severe toxicity and response to treatment.

Our data also suggested that grade 3 and 4 adverse events correlated with a better radiological response, a widely accepted marker for treatment efficacy. While data correlating toxicity with radiological response is scarce, in one retrospective analysis of 119 patients with metastatic melanoma treated with anti-CTLA-4 therapy including ipilimumab and tremelimumab, radiological manifestations of immune related adverse events were significantly associated with disease control and improved clinical response [17]. The disease control rate was 55% for the group with radiological manifestations of immune-related adverse events. In contrast, only 10% of patients without radiological manifestations experienced disease control.

The NCI phase I data also showed that low grade toxicity (grade 1 and 2) was moderately associated with the length of ipilimumab therapy, while high grade toxicity (grade 3 and 4) was not associated with duration of treatment. These findings are consistent with the findings of a retrospective analysis of 40 patients in a phase III ipilimumab plus glycoprotein 100 peptide vaccine (gp100) study who later underwent prolonged therapy via re-exposure to ipilimumab [3, 18]. Not only did some patients experience a longer and more effective response after retreatment, side effects were similar to what was observed during initial therapy with no new toxicities. Most of the immune related adverse events were mild (grade 1 and 2), affected the skin and gastrointestinal tract, and were managed with established treatment algorithms. No grade 4 or 5 toxicities occurred in the patients re-treated. Furthermore, the study concluded that toxicities seen initially did not predispose to future toxicity during re-exposure treatment, and toxicity was not cumulative between treatments. In another study consisting of 855 patients treated with ipilimumab, 51 patients were re-treated upon disease progression [19]. Of these patients, 22% experienced an adverse event of any grade during the retreatment period, and these were generally mild-to-moderate and similar in frequency compared to induction. Six percent of the patients experienced a grade 3 or 4 adverse event related to ipilimumab upon retreatment. No new types of toxicity occurred.

Our data also suggests that pretreatment lymphocyte count, LDH and albumin levels do not correlate with toxicity. While phase III data that evaluates these parameters are not available, smaller retrospective analyses evaluate these variables as potential biomarkers to predict ipilimumab efficacy. One study assessed 209 advanced melanoma patients treated with ipilimumab and concluded that the occurrence of adverse events did not correlate with baseline LDH nor relative lymphocyte count [16]. In a retrospective analysis of 183 patients treated with ipilimumab, LDH and neutrophil-to-lymphocyte ratio could differentiate patients into high, moderate and low risk of death, but was not associated with toxicity [12]. Another analysis conducted on 47 advanced melanoma patients also demonstrated a significant association between higher LDH and poor prognosis, but no association between LDH and toxicity [20].

Finally, no associations were found between adverse events and ECOG performance status in our phase I database analysis. Our data did not exhibit trends that would suggest a worse side effect profile in patients with poor performance status, as there was not a significant difference between toxicity in ECOG PS 0 versus 1/2 patients (patients with ECOG PS > 2 were not included in NCI Phase I clinical trials). Unlike chemotherapy, where tolerability depends on baseline performance status of the patient as therapy is taxing on healthy tissue, immunotherapy side effects are often auto-immune driven which are unpredictable and theoretically unrelated to baseline performance status. Although minimal prospective data exist on the effect of ECOG performance status in response to ipilimumab, retrospective analyses have suggested associations between ECOG performance status and survival but not with toxicity. For example, a study of 58 metastatic melanoma patients treated with ipilimumab demonstrated that performance status > 1 was associated with worse survival, as shown by univariate analysis. However, no conclusions were made on an association between toxicity and performance status. In a study consisting of 129 metastatic melanoma patients treated with ipilimumab, univariate analysis showed that a better baseline ECOG performance status (0 versus 1) was associated with improved response to treatment [12]. Again, no conclusions could be made to correlate ECOG performance status with toxicities observed during treatment.

## MATERIALS AND METHODS

For this retrospective study, two authors queried the NCI’s Integrated Platform for Agents and Diseases (IPAD) database (version 6.1.0, Rockville, MD) in May 2018 to determine the number of ipilimumab immunotherapy trials alone or in combination in the NCI portfolio at the time. The clinical trials were sorted using search terms of “ipilimumab,” “Phase 1 or 1/2,” and “complete, closed to accrual,” or “closed to accrual and treatment.” Once trials were identified, a single reviewer abstracted data from anonymized patient experience adverse reports in tabular format. Only ipilimumab-attributed adverse events like symptoms or signs of toxicity, as well as decreased hematological cell counts, blood electrolytes, or proteins were analyzed in this study; therefore, the prevalence of toxicities reported here may differ from primary research publications [21–31]. Events repeating across consecutive cycles were considered a single event for this analysis. Patient demographic, Eastern Cooperative Oncology Group (ECOG) performance status scale score, tumor characteristics and treatment regimens were also abstracted (Table 2). When available, duration of ipilimumab exposure, radiographic responses, disease relapse events and mortality status were recorded. A second reviewer independently provided a data quality-check. The research presented in this article involved the collection and analysis of existing data, documents and records that were publicly available. This research is regarded as exempt from Institutional Review Board oversight.

Categorical data were analyzed using Fisher exact test or Pearson chi-square test and continuous data were analyzed using Wilcoxon rank-sum test (α = 0.05). Correlation between covariates and outcome were presented using Pearson correlation coefficient.

## CONCLUSIONS

Ipilimumab-attributed grade 3 or 4 toxicity is associated with radiological response in this limited series of 11 early phase I clinical trials. Pre-trial performance status, pre-treatment lymphocyte count, lactate dehydrogenase and albumin levels were not associated with ipilimumab-attributed toxicity. The number of added antitumor agents to ipilimumab treatment raised the likelihood of treatment-related adverse events, and duration of treatment was associated with low grade rather than higher grade toxicity.
